# Extracellular Vesicles Derived from *Acholeplasma laidlawii* PG8

**DOI:** 10.1100/tsw.2011.109

**Published:** 2011-05-26

**Authors:** Vladislav M. Chernov, Olga A. Chernova, Alexey A. Mouzykantov, Irina R. Efimova, Gulnara F. Shaymardanova, Elena S. Medvedeva, Maxim V. Trushin

**Affiliations:** ^1^ Kazan Institute of Biochemistry and Biophysics, Russian Academy of Sciences, Kazan, Russia; ^2^ Kazan Federal University, Kazan, Russia

**Keywords:** mycoplasmas, Acholeplasma laidlawii, ultramicroforms, membrane vesicles, morphology, DNA, proteins, mutagenicity

## Abstract

Extracellular vesicle production is believed to be a ubiquitous process in bacteria, but the data on such a process in Mollicutes are absent. We report the isolation of ultramicroforms – extracellular vesicles from supernatants of *Acholeplasma laidlawii* PG8 (ubiquitous mycoplasma; the main contaminant of cell culture). Considering sizes, morphology, and ultrastructural organization, the ultramicroforms of *A. laidlawii* PG8 are similar to membrane vesicles of Gram-positive and Gram-negative bacteria. We demonstrate that *A. laidlawii* PG8 vesicles contain genetic material and proteins, and are mutagenic to lymphocytes of human peripheral blood. We show that *Mycoplasma gallisepticum* S6, the other mycoplasma, also produce similar structures, which suggests that shedding of the vesicles might be the common phenomenon in Mollicutes. We found that the action of stress conditions results in the intensive formation of ultramicroforms in mycoplasmas. The role of vesicular formation in mycoplasmas remains to be studied.

## INTRODUCTION

Mollicutes (mycoplasmas) are widely distributed as pathogens or commensal organisms of a wide range of plants and animals, including invertebrates and vertebrates[1]. The main motivation for working with these bacteria has been their association with diseases of human beings and economically important diseases of agricultural animals and plants[2]. Additionally, representatives of the class Mollicutes were recognized as contaminants of cell cultures[3] and media products applied in biotechnology[4]. In turn, addressing the applied problems connected with these bacteria is not possible without a fundamental knowledge of their biology, mechanisms of their adaptation to environmental conditions, and realization of their virulence.

It is known that microorganisms in stressful circumstances are able to transform into a new physiological state that is accompanied by alterations in cell morphology and molecular biology[5]. Some of these changes were also found in mycoplasmas[6,7,8,9]. It was recently reported that membrane vesicles – spherical, closed membranous nanostructures (with diameters from 20 to 250 nm) produced by cells of microorganisms – may play a significant role in adaptation to the environmental conditions and realization of virulence in bacteria[10]. Membrane vesicles were described for archaebacteria, Gram-positive bacteria, and Gram-negative bacteria[11,12,13]. It was suggested that membrane vesicles mediate the universal way for protein secretion and intercellular interactions in all organisms – pro- and eukaryotes[14]. However, experimental data on the investigation of membrane vesicles in mycoplasmas are absent.

We previously reported that a culture of *Acholeplasma laidlawii* PG8 – a ubiquitous mycoplasma and the main contaminant of cell cultures – contains (apart from typical mycoplasma cells) ultramicroforms with sizes less than found in natural nanobacteria – 200 nm[7]. Clarification of the nature of *A. laidlawii* PG8 ultramicroforms and their possible relationship with bacterial membrane vesicles suggests a complex investigation of the corresponding structures.

The present work is devoted to the investigation of *A. laidlawii* PG8 ultramicroforms from the viewpoint of their morphologic, ultrastructural, molecular-genetic, and pathogenic features.

## MATERIALS AND METHODS

*Acholeplasma laidlawii* PG8 and *Mycoplasma gallisepticum* S6 strains were obtained from the N.F. Gamalei Research Institute of Epidemiology and Microbiology (Moscow, Russia). *A. laidlawii* PG8 and *M. gallisepticum* S6 cells were cultivated for 2 days at 37°C on Edward’s medium (tryptose, 2% [w/v]; NaCl, 0.5% [w/v]; KCl, 0.13% [w/v]; Tris base, 0.3% [w/v]; serum of horse blood, 10% [w/v]; fresh yeast extract, 5% [w/v]; glucose solution, 1% [w/v]; benzylpenicillin [500,000 IE/ml], 0.2% [w/v]) to obtain the control cells. To obtain experimental cells, glucose, serum of horse blood, and yeast extract were eliminated from the Edward’s medium; microorganisms were continuously kept under this condition as previously described[7]. To prepare samples for atomic force microscopy (AFM) studies, 1 mL of the corresponding *A. laidlawii* PG8 and *M. gallisepticum* S6 cells were centrifuged at 12,000 rpm for 20 min at room temperature. The pellet was resuspended in 1 mL of PBSx1 (pH = 7.2). Then, cells were again centrifuged at 12,000 rpm for 15 min at room temperature and repeatedly resuspended in 0.5 mL of the same buffer. The prepared cells (5 μL) were placed onto the mica (Advanced Technologies Center, Moscow, Russia) with the upper layer removed. Cells were air dried and then rinsed twice with redistilled water, and after each rinsing, the samples were air dried in both instances.

AFM imaging was performed with a Solver P47H atomic force microscope (NT-MDT, Moscow, Russia) operating in the tapping mode using fpN11S cantilevers ($r \leq 10$ nm, Advanced Technologies Center, Moscow, Russia). The height, Mag (signal from lock-in amplifier), RMS (signal from RMS detector), and phase (signal from the phase detector) were performed with the Nova 1.0.26 RC1 software (NT-MDT). The scan rate was 1 Hz. Image resolution was $512 \times 512$. Numerical data are presented as mean ± SE.

Isolation of ultramicroforms from *A. laidlawii* PG8 culture (poststationary growth phase) was performed according to Kolling and Matthews[15], with some modifications. Cell were precipitated with centrifugation at 15,000 *g* for 40 min. Supernatant was resuspended in phosphate-salt buffer and then filtered through a sterile filter (Sartorius Minisart, France) with a pore size of 200 nm.

Transmission electron microscopy (TEM) was done with a JEM-1200EX microscope (Japan) according to Chernov et al.[7]. The negative-staining TEM was performed as described in Rivera et al.[16].

PCR primers were constructed in NSF “Litekh” (Moscow, Russia) using the nucleotide sequences of *A. laidlawii* PG8-A genes (GenBank accession number NC_010163): *ftsZ* (Ala1F 5'-ggtttttggatttaacgatg-3' Ala1R 5'- gcttccgcctcttttattt-3'), *dnaK* (Ala2F 5'-gtgaagacaatggcgaagac-3' Ala2R 5'- tgcatcagttgctccgttt-3'), *mutS* (Ala3F 5'- atgaagggagcatattatgg-3' Ala3R 5'- tccatctatttaacctcctt-3'), *dnaA* (Ala4F 5'-caaacagcacattatggcag-3' Ala4R 5'-caatcgagtcaacagcaaac-3'), *parE-parC* (Ala5F 5'- cgctacgtggtaaggtgatt-3' Ala5R 5'-caccatctatggaaccattg-3'), *pdhA* (Ala6F 5'-gatcaaaacggtaaagtggt-3' Ala6R 5'-tctttttccattgcttggtc-3'), *fbpA* (Ala7F 5'-gaaatacctagaaggtggca-3' Ala7R 5'-taatcaatggcacgtttacc-3' and

Ala8F 5'-aaacaacttgaccaagcaca-3' Ala8R 5'-ccaccattgtttctaactgc-3'), *pdhC* (Ala9F 5'-aaagcaagaccataaggagg-3' Ala9R 5'-tggagcctgtgtttgttga-3' and Ala10F 5'-cgaacaaggtagagttatgaaag-3' Ala10R 5'-agttggaatacaaccgtgg-3'), *pnp* (Ala12F 5'-taacggttcaagttctcaag-3' Ala12R 5'-catccagtatcatcattggc-3'), *trx* (trx3F 5'- ggcaagaaggcgaaggttt-3' trx3R 5'-gcctgtggtgtcatctgtatc-3'), spacer 16S-23S of ribosome operon (A16LF 5'-ggaggaaggtggggatgacgtcaa-3' A23LR 5'-ccttaggagatggtcctcctatcttcaaac-3').

PCR was performed in the following regime: for primers Ala1–5, 95°C, 3 min (95°C, 30 sec; 52°C, 90 sec; 72°C, 60 sec) (30 cycles); 72°C, 10 min. For primers Ala6–12, 95°C, 3 min (95°C, 15 sec; 55°C, 10 sec; 72°C, 10 sec) (20 cycles); 72°C, 10 min. For primers trx3, 95°C, 3 min (95°C, 30 sec; 63°C, 30 sec; 72°C, 10 sec) (35 cycles); 72°C, 5 min. For primers A23LR, 95°C, 3 min (95°C, 5 sec; 63°C, 5 sec; 72°C, 20 sec) (30 cycles); 72°C, 5 min.

Prior to two-dimensional electrophoresis (2DE), pellets of cells and ultramicroforms were treated with CHAPS and a mix of nucleases (GE Healthcare, U.K.). Then proteins were dissolved in buffer containing 8 M urea, 2 M thiourea, 5% ampholines (pH 3–10), 80 mM McClelland's reagent, 16.7% solution of 30% CHAPS (GE Healthcare), and 10% NP 40. 2DE protein separation was performed as described[17].

1DE protein separation was performed according to Laemmli[18] in 12% polyacrylamide gel in tris-glycine buffer in the following regime: concentrating gel 80 V, 80 mA, 30 min; separating gel 120 V, 80 mA, 5 h. Gels were stained with silver nitrite.

Karyotypic analysis of lymphocytes of human peripheral blood was done according to Chernova et al.[19]. These human cells were cultured in the presence of *A. laidlawii* PG8 cells (200 μL of mycoplasma cells [initial titer of $10^7$ CFU] resuspended in phosphate-salt buffer was added to 0.3 mL of lymphocytes) and ultramicroforms of the mycoplasma (50 μL of resuspended in phosphate-salt buffer was added to 0.3 mL of lymphocytes).

Statistical treatment of results was made with MS Excel software; general value for probability was calculated with the exact formula of F-distribution[20]; $p < 0.05$ was considered significant to indicate differences.

## RESULTS AND DISCUSSION

According to AFM data (Fig. 1), *A. laidlawii* PG8 culture contains (except for typical mycoplasma cells with 400- to 600-nm diameter) ultramicroforms with diameters of 70–90 and 110–120 nm (Table 1). Ultramicroforms could be found near the cells (empty arrow) and at the cell surfaces (black arrow). It should be noted here that a formation of the ultramicroforms was detected only at the surface of *A. laidlawii* PG8 cells with sizes >300 nm. Production of ultramicroforms is a process that occurs during normal growth of *A. laidlawii* PG8. Meanwhile, in stress conditions (starvation), a number of ultramicroforms proved to increase (Fig. 2). The overwhelming majority of ultramicroforms tended to interact with each other and form tree-like structures at the surface of mica.

According to TEM data (Fig. 3), *A. laidlawii* PG8 ultramicroforms are a heterogeneous group on their electronic density. We observed *A. laidlawii* PG8 ultramicroforms that are spherical structures with bilayered membranes and electron-dense luminal contents. Considering sizes, morphology, and ultrastructural organization, the ultramicroforms of *A. laidlawii* PG8 are similar to membrane vesicles of Gram-positive and Gram-negative bacteria (Fig. 4). We found that *M. gallisepticum* S6, the other mycoplasma, also produces similar structures (Fig. 2B), which suggests that the shedding of the vesicles might be the common phenomenon in Mollicutes.

It was shown that membrane vesicles in bacteria are composed of lipopolysaccharides, lipids, soluble or membrane-associated proteins, genetic material, toxins, and some other factors associated with virulence[12,13,14]. The vesicle production was investigated mainly in Gram-negative bacteria[12], and recently the process was detected in Gram-positive bacteria phylogenetically related to mycoplasmas[13]. Heterogeneity among membrane vesicles was shown[16]. For example, the size of membrane vesicles in

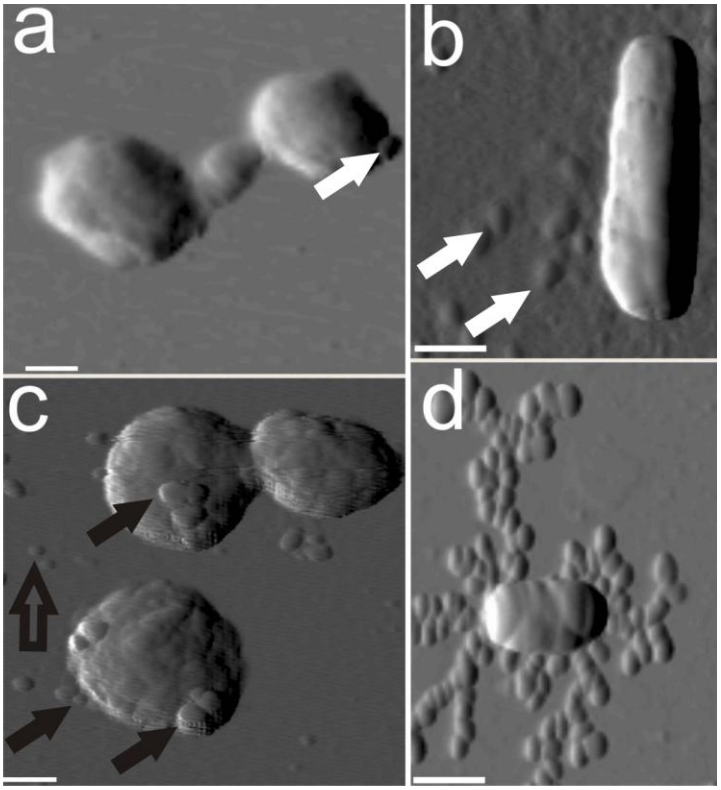

**FIGURE 1**. *A. laidlawii* PG8 (a, c) and *M. gallisepticum* S6 (b, d) cells cultivated in favorable (a, b) and unfavorable (c, d) conditions. Bars correspond to 0.2 µm.

**TABLE 1**
**The Percentage of Ultramicroforms with the Definite Sizes Found in *A. laidlawii* PG8 and *M. gallisepticum* S6**

| Organism | Diameter of Ultramicroforms (nm) | | | | | | | | | |
|---|---|---|---|---|---|---|---|---|---|---|
| | 20–29 | 30–39 | 40–49 | 50–59 | 60–69 | 70–79 | 80–89 | 90–99 | 100–109 | 110–119 |
| *A. laidlawii* PG8 | ND | ND | ND | ND | ND | 48 | 4 | ND | ND | 48 |
| *M. gallisepticum* S6 | 4 | 60 | 24 | 8 | 4 | ND | ND | ND | ND | ND |

ND – not detected.

Gram-positive *Bacillus anthracis* ranged from 50 to 150 nm[16] and from 20 to 100 nm in *Staphylococcus aureus*[13]. It is interesting to note that a majority of membrane vesicles in *S. aureus* was found in diapason of 30–50 nm, as we also observed for ultramicroforms in *M. gallisepticum* S6 (Fig. 2). As to *A. laidlawii* PG8, we detected two size maximums of vesicles in the range of 70–80 and 110–120 nm. These size vesicles are probably different in their content and functions.

Revealing the nature of *A. laidlawii* PG8 ultramicroforms needs the isolation of the corresponding mycoplasma structures and investigation of their content. Fig. 4 presents a micrograph of ultramicroforms isolated from the supernatants of *A. laidlawii* PG8. This picture is similar to those for extracellular vesicles from supernatants of Gram-positive and Gram-negative bacteria[13,16,21].

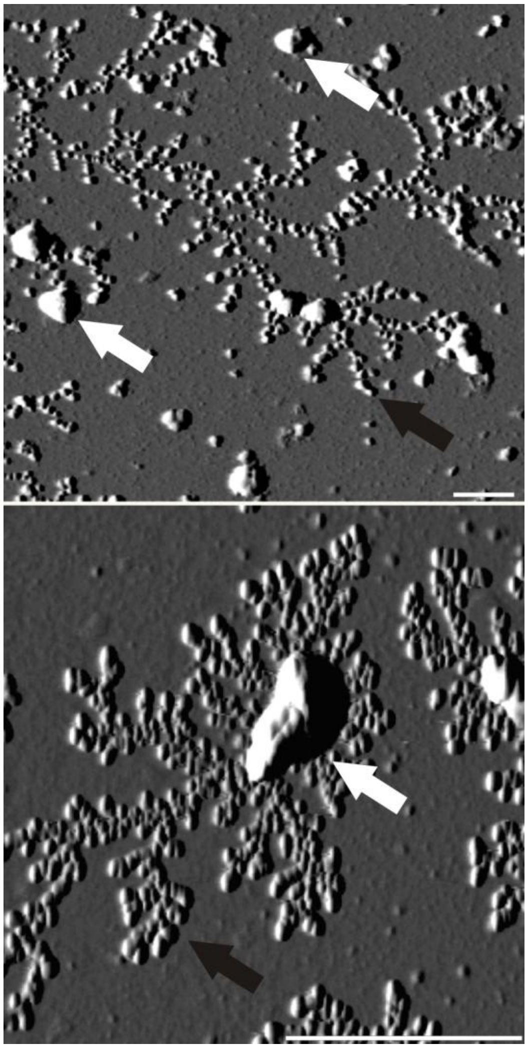

**FIGURE 2.** Representation of ultramicroforms as tree-like structures found in *A. laidlawii* PG8 (top) and *M. gallisepticum* S6 (bottom) cells cultivated in unfavorable conditions. White arrows = "typical" mycoplasma cells, black arrows = ultramicroforms. Bars correspond to 1 µm.

Fig. 5 presents the results of PCR analysis of *A. laidlawii* PG8 cells and ultramicroforms. The obtained data confirmed that ultramicroforms of the mycoplasma contain genetic material. The absence of PCR signals regarding the nucleotide sequences of some genes (*pdhA*, *fbpA*, *pdhC*, *pnp*, *trx*, *rrnA*, *rrnB*) might be mediated by the absence of the corresponding nucleotide sequences or alteration of their matrix features in ultramicroforms[22].

Data of Figs. 6 and 7 confirmed that A. laidlawii PG8 ultramicroforms contain proteins, including soluble proteins. SDS-PAGE analysis of proteins derived from ultramicroforms of A. laidlawii PG8 demonstrated that vesicles of the mycoplasma have distinct protein patterns compared with other subcellular localizations (Fig. 6). 2DE allows us to visualize about 40 soluble proteins derived from the mycoplasma ultramicroforms (Fig. 7B) that are comparable to data for membrane vesicles from other bacteria[23,24]. MS-based proteomics studies on the ultramicroforms of A. laidlawii PG8 may be a powerful approach to decode their components.

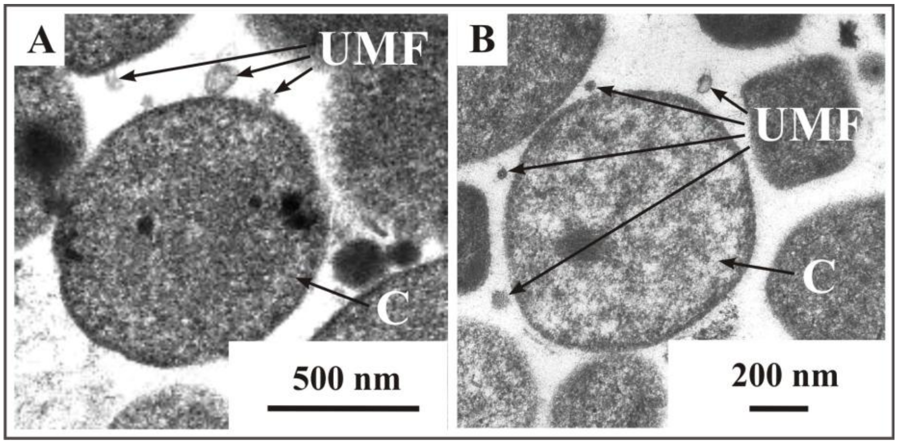

**FIGURE 3.** Cells and ultramicroforms of *A. laidlawii* PG8. C = “typical” mycoplasma cells; UMF = ultramicroforms.

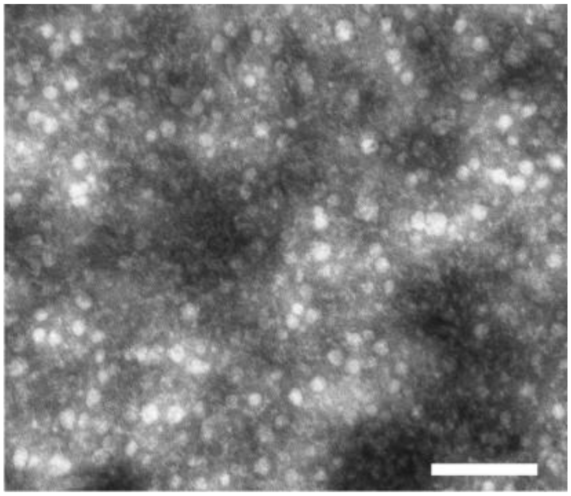

**FIGURE 4.** Negative-staining TEM of purified ultramicroforms of *A.laidlawii* PG8 after buoyant density-gradient centrifugation. Bar corresponds to 100 nm.

Bacterial membrane vesicles contain toxins and may show genotoxic features toward cells of eukaryotes. It was found in our study that *A. laidlawii* PG8 ultramicroforms may display mutagenicity toward lymphocytes of human peripheral blood. For example, the combined cultivation of lymphocytes of human peripheral blood with *A. laidlawii* PG8 ultramicroforms resulted in chromosomal aberrations in 95% of cells, but 15 and 2% after cultivation with typical mycoplasma cells and in controls (spontaneous mutations), respectively. Multiple polysomies were dominant among the observed aberrations (Fig. 8). Bacterial vesicles may represent potent vehicles for toxin transmission to host cells. In this connection, identification of proteins and other components of *A. laidlawii* PG8 ultramicroforms seems realistic from the viewpoint of potential mutagenic factors secreted via vesicles.

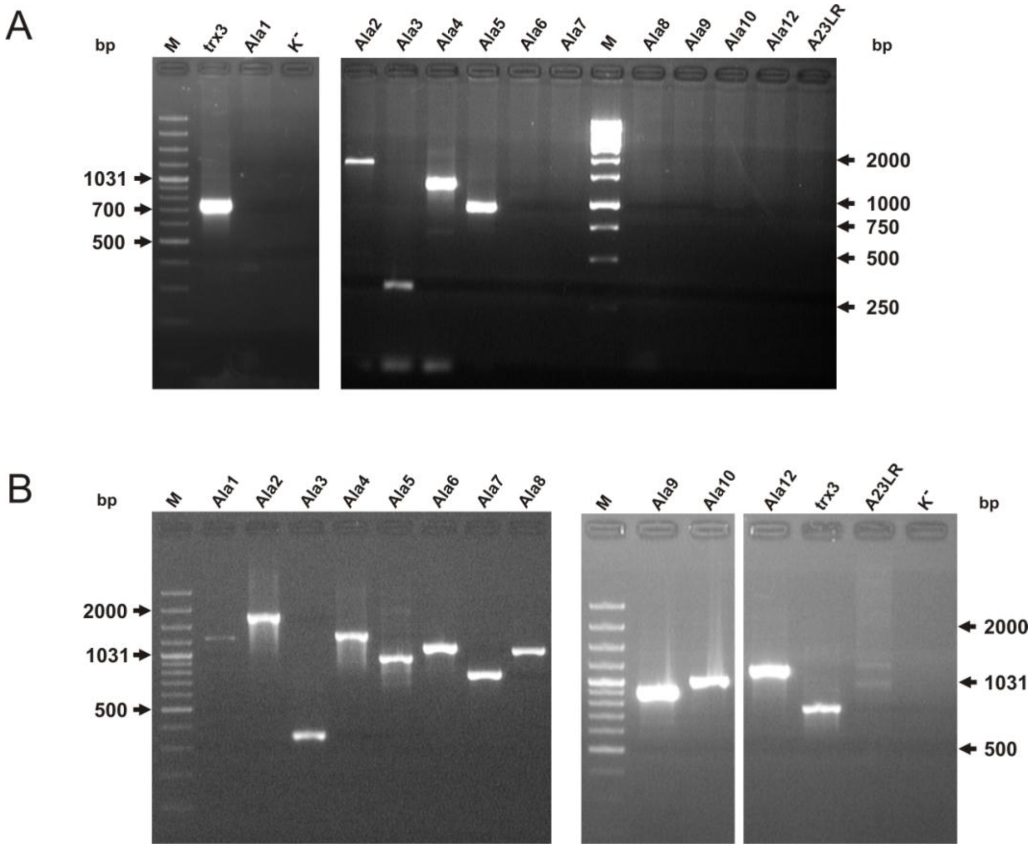

**FIGURE 5.** Electropherogram of amplification products for DNA nucleotide sequences from *A. laidlawii* PG8 ultramicroforms (A) and cells (B). M = marker of molecular weight; K = negative control. Primers are indicated above the gels.

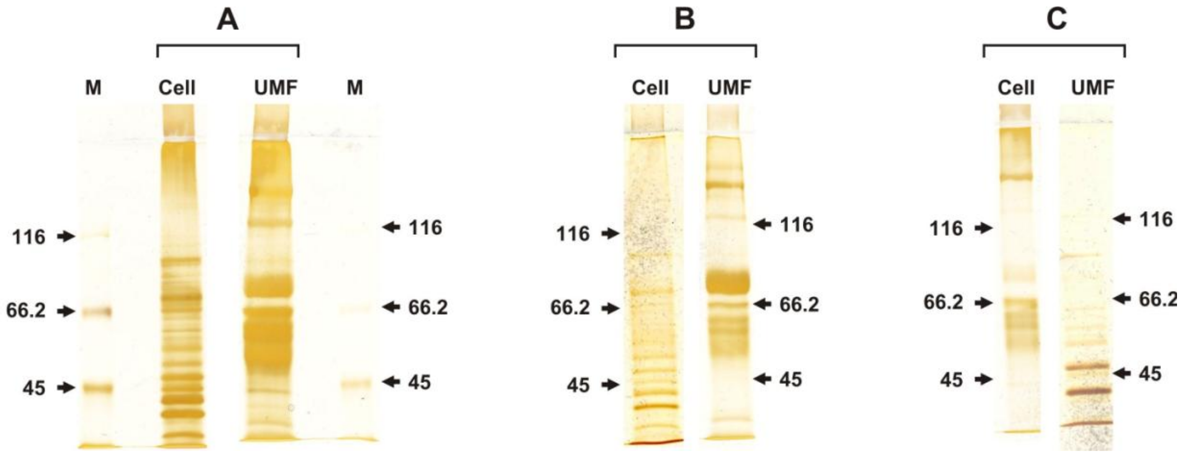

**FIGURE 6.** SDS-PAGE of proteins *A. laidlawii* PG8 cells and ultramicroforms (UMF) derived from lysates of whole cells (A), membrane (B), and cytoplasm. M = marker of molecular mass (kDa).

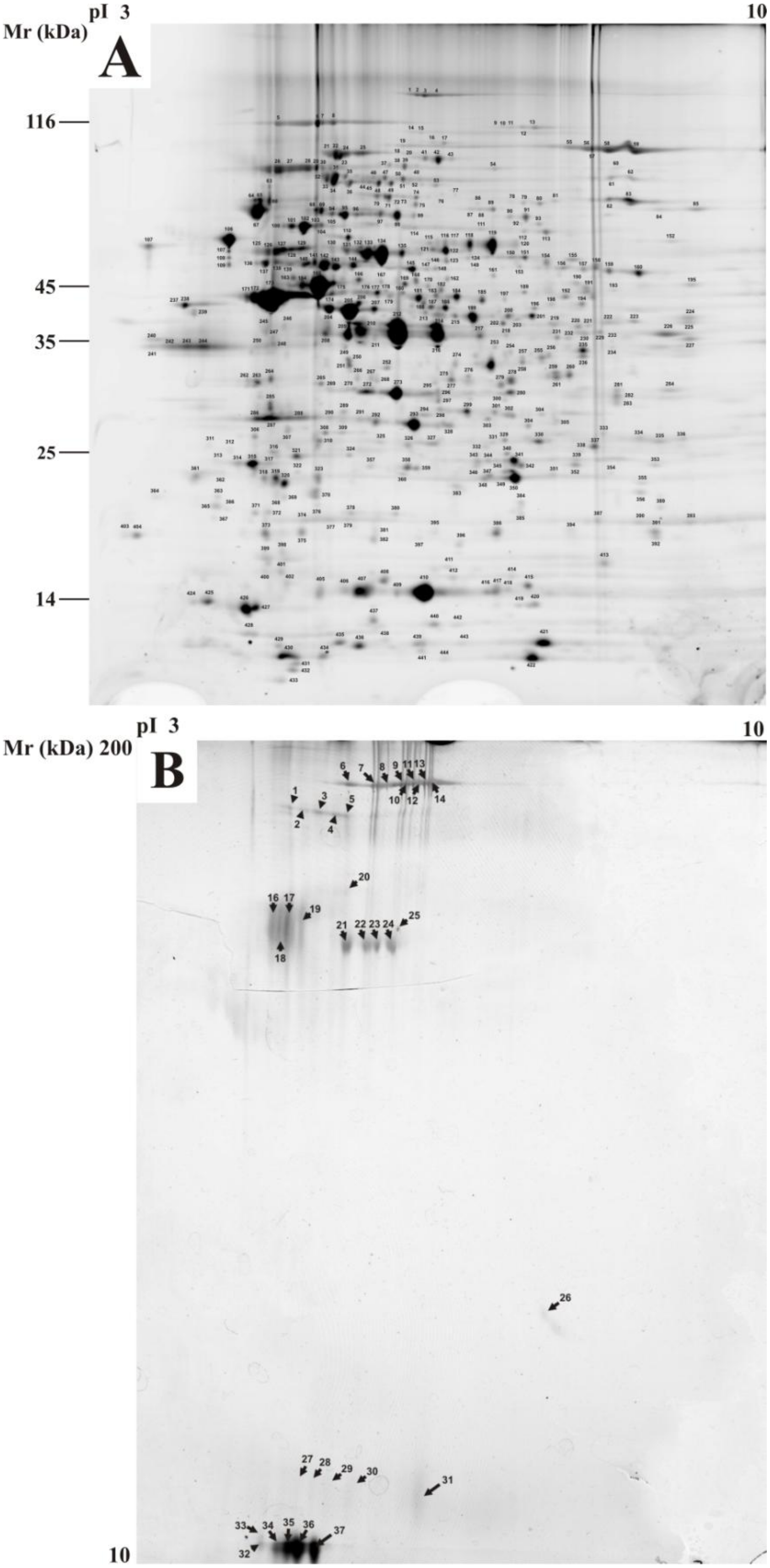

**FIGURE 7.** 2DE of soluble proteins from cells (A) and ultramicroforms (B) of *A. laidlawii* PG8.

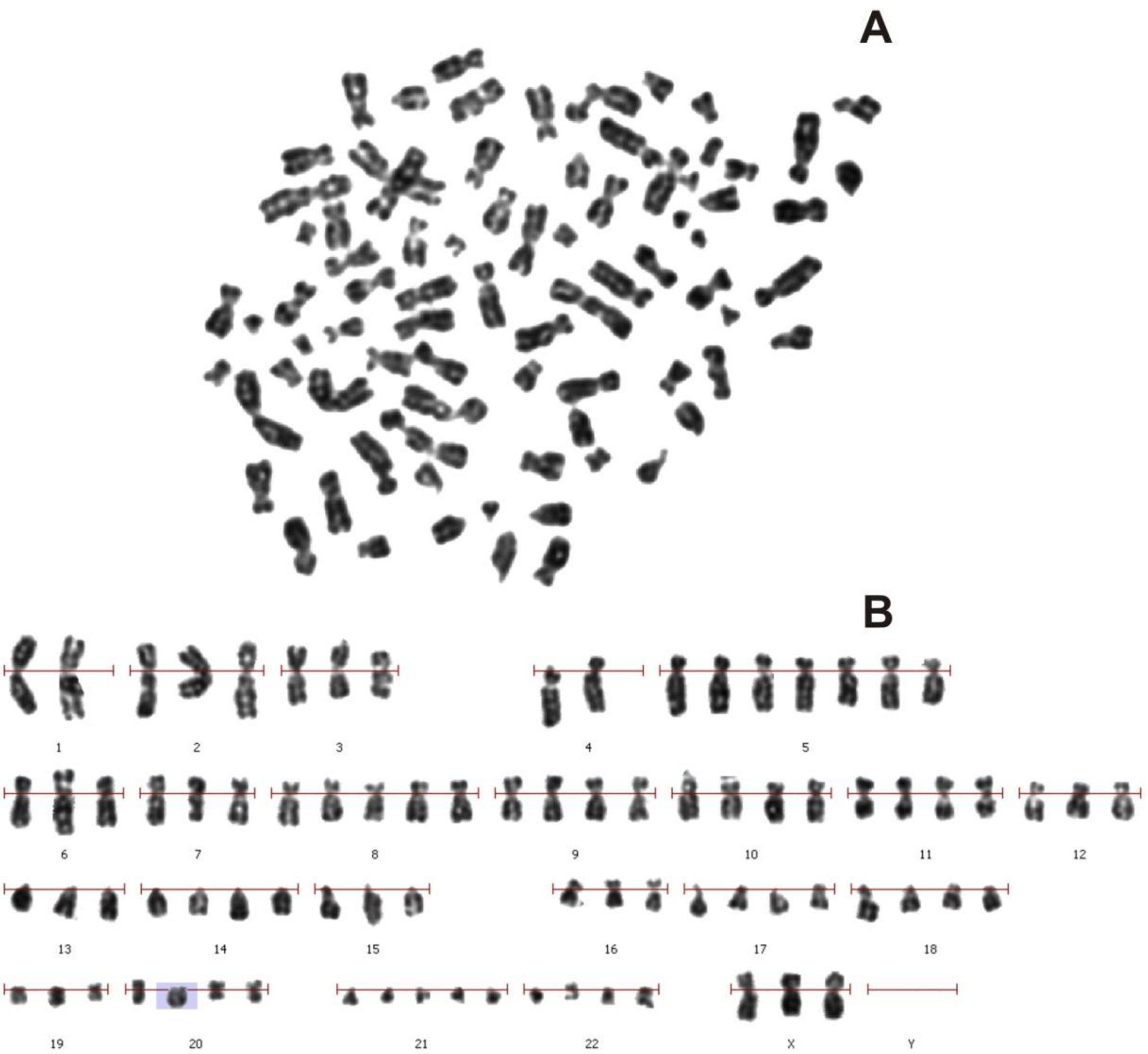

**FIGURE 8.** Multiple polysomy induced by ultramicroforms in lymphocytes of human peripheral blood. (A) Metaphase plate; (B) karyogram.

It was detected in our studies that in stress conditions, a number of ultramicroforms was increased in *A. laidlawii* PG8 as well as in *M. gallisepticum* S6[25,26,27]. At that, significant changes in the physiology of the microorganisms as well in matrix features of morphometric parameters of DNA were found[8,28]; changes in the virulence of the bacteria were also detected[7,25]. Production of *A. laidlawii* PG8 ultramicroforms is probably involved in the adaptation of the mycoplasma to unfavorable factors and realization of virulence in stress conditions. Most likely, understanding the logic of life for the smallest prokaryotes and development of the efficient control of the associated infections needs a broad investigation of biogenesis and functions of mycoplasmal ultramicroforms – the extracellular vesicles.

Thus, in this work, some data on morphologic, ultrastructural, and molecular-genetic aspects of *A. laidlawii* PG8 ultramicroforms were presented for the first time; the data allow us to consider the ultramicroforms as membrane vesicles mediating in bacteria protein secretion, intercellular interactions, and pathogenesis. However, the role of the structures in mycoplasmas – the smallest wall-less prokaryotes – remains to be studied.

## ACKNOWLEDGMENTS

This work was supported by grants of the President of the Russian Federation (MK-3372.2009.4 and MK-4894.2010.4), a grant of the Russian Fund for Basic Research (Project #11-04-01406a), Scientific School (Project #6992.2010.4, Head – Academician A.N. Grechkin), and Government contract #02.740.11.0391. We thank Prof. V.V. Salnikov for help with the TEM investigation, physician-geneticist L.R. Samoilova for help with karyotypic analysis, and Dr. O.A. Konovalova from Kazan Federal University for help with the AFM research.
